# Long-term outcome of patients with liver cirrhosis admitted to a general intensive care unit

**DOI:** 10.1186/s13613-017-0257-6

**Published:** 2017-04-04

**Authors:** Alex Warren, Charlotte R. Soulsby, Alex Puxty, Joseph Campbell, Martin Shaw, Tara Quasim, John Kinsella, Joanne McPeake

**Affiliations:** 1 Academic Unit of Anaesthesia, Pain and Critical Care, University of Glasgow, Room 2.73, Level 2, New Lister Building, Glasgow Royal Infirmary, 10-16 Alexandra Parade, Glasgow, Scotland G31 2ER UK; 2 Intensive Care Unit, NHS Greater Glasgow and Clyde, 84 Castle Street, Glasgow, Scotland G4 OSF UK; 3 Medical Physics, NHS Greater Glasgow and Clyde, Level 2, New Lister Building, Glasgow Royal Infirmary, 10-16 Alexandra Parade, Glasgow, Scotland G31 2ER UK

**Keywords:** Critical care, Cirrhosis, Scoring tools, Child–Pugh, Lactate

## Abstract

**Objectives:**

The prevalence of liver cirrhosis is increasing, and many patients have acute conditions requiring consideration of intensive care. This study aims to: (a) report the outcome at 12 months of patients with cirrhosis admitted to ICU, (b) identify factors predictive of long-term mortality and (c) evaluate the ability of scoring systems to predict long-term outcome.

**Design:**

Observational cohort study.

**Setting:**

General adult critical care unit in a UK teaching hospital.

**Patients:**

Eighty-four patients admitted to critical care between June 2012 and December 2013.

**Primary outcome measures:**

Cumulative survival at ICU discharge, hospital discharge and 12 months.

**Results:**

Eighty-four patients with diagnosed cirrhosis were followed up at 12 months. Clinical variables collected at ICU admission were entered into a multivariate regression analysis for mortality and eight predetermined scoring systems calculated. Cumulative survival at ICU discharge, hospital discharge and 12 months was 64.8, 47.1 and 44.1%, respectively. Twelve months of cumulative survival in patients with Child–Pugh class A was 100%, class B was 50% and class C was 25% (log rank *p* = 0.002). Independent predictors of mortality at 12 months were lactate, bilirubin, PT ratio and age. The Child–Pugh + Lactate score was modified to produce an objective score comprising Albumin, Bilirubin and Clotting (PT ratio) added to serum lactate concentration in mmol L^−1^ (ABC + Lactate). This score was the best predictor of 12-month survival, with an AUC of 0.83. A proposed classification by ABC + Lactate score was highly significant (*p* = 0.001), with those in the highest class having ICU mortality of 75% and hospital and 12-month mortality of 93%.

**Conclusions:**

Patients with cirrhosis admitted to ICU have high initial mortality but low mortality after hospital discharge. Child–Pugh class at ICU admission predicts outcome at 12 months. The ABC + Lactate classification system may be useful in identifying critically ill cirrhotic patients with very high long-term mortality.

## Background

Liver disease is the third most common cause of premature death in the United Kingdom (UK) [1], and patients with liver cirrhosis now comprise between 2.6 and 15% of admissions to intensive care units (ICUs) in the UK [2–5]. Though survival in this patient group has historically been very poor, with reported hospital mortality as high as 89% [6–8], it has recently demonstrated that short-term survival is now improving [9–11].

Whilst this population has been well-studied with respect to short-term outcome [3–5, 10–14], relatively few studies have reported on long-term outcomes of critically ill cirrhotic patients. A 2006 study from a Scottish transplant centre demonstrated 12-month mortality of 81% in ICU admissions with decompensated alcoholic cirrhosis [4]; other non-UK transplant centres have reported 6-month mortality of between 62 and 71% [15–17]. Long-term outcome data from cirrhotic patients admitted to general ICUs are also scarce, with one European centre reporting 6-month mortality of 60% amongst all admissions, another reporting 12-month mortality of 82% and one UK centre reporting 12-month mortality of 59% [3, 18, 19].

It is difficult to predict which patients will benefit from ICU admission, and the use of scoring systems has been proposed to inform clinical decision-making. The most widely used method for assessing patients with chronic liver disease is the Child–Pugh score; however, this has been found to poorly predict short-term outcome in cirrhotic patients admitted to ICU [5, 11, 12, 19–21]. Many studies have found general critical illness scores, such as Acute Physiology, Age and Chronic Health Evaluation II (APACHE II) and Sequential Organ Failure Assessment (SOFA) to be superior to liver-specific scores such as Child–Pugh, the Model for End-Stage Liver Disease (MELD) and the UK Model for End-Stage Liver Disease (UKELD) [5, 11, 15, 18, 21]. Novel scores have also been designed for the acutely ill cirrhotic population, such as the Royal Free Hospital (RFH) score [11] and the Chronic Liver Failure-Sequential Organ Failure Assessment (CLIF-SOFA) [22]. Increased serum lactate has been widely found to predict short-term mortality in critically ill cirrhotic patients [5, 11, 12, 16, 23], leading to the development of scores incorporating lactate such as SOFA-Lactate [16]. Child–Pugh + Lactate was also found to be the best performing score at predicting ICU mortality in a previous study from this centre [5].

Despite this significant body of research, none of these scores have been validated with respect to long-term outcomes of critically ill cirrhotic patients. The aims of our study were: (a) to report the 12-month outcome of cirrhotic patients admitted to a general ICU; (b) to identify any factors predictive of survival at 12 months; and (c) to compare the ability of scoring systems to predict long-term outcome in this population.

## Methods

An observational cohort study of patients with liver cirrhosis admitted to the adult ICU at Glasgow Royal Infirmary was undertaken between June 2012 and December 2013. We have previously reported on the acute mortality of this population [24]. This unit is a 20-bed mixed surgical and medical critical care unit within a university hospital which does not provide liver transplant services. Patients were enrolled if they had received ‘Level 3’ care in the unit, defined as multiple organ support and/or mechanical ventilation. A diagnosis of cirrhosis was made if the patient had a positive liver biopsy on record, or if they had clinical features of cirrhosis plus any of the following: evidence of portal hypertension, ascites, encephalopathy or signs of liver cirrhosis on ultrasound examination. Patients with all aetiologies of liver cirrhosis were included, and all diagnoses were validated by a second independent clinician. Patients who were considered eligible for acute liver transplantation were referred to the regional transplant centre.

Data were collected prospectively from the Philips Intellivue Clinical Information Portfolio (Philips Medical Systems, Eindhoven, the Netherlands) and WardWatcher (Critical Care Audit Ltd., Yorkshire, UK) computerised patient record systems at the time of ICU admission. Demographic data collected included: age, gender, postcode, indication for ICU admission and aetiology of cirrhosis. The postcode was used to calculate the Scottish Index of Multiple Deprivation (SIMD) quintile for each patient, with those in the lowest quintile considered socially deprived. Clinical data were taken from the first recorded value following the documented time of ICU admission and included sodium, potassium, urea, arterial lactate, bilirubin, albumin, creatinine, total white cell count, prothrombin time (PT) ratio, platelet count, ratio of arterial oxygen partial pressure to fraction of inspired oxygen (PaO_2_/FiO_2_ ratio), and ascites and encephalopathy grades. Encephalopathy was graded using the West Haven criteria prior to intubation or sedation.

Based on the previously described literature, several scoring systems were chosen for inclusion in this study and were calculated at admission as previously described by their authors. These comprised Child–Pugh [25], MELD, UKELD, APACHE II and SOFA [25–29]. In addition to these established systems, the novel RFH, CLIF-SOFA, SOFA-Lactate and Child–Pugh + Lactate were calculated [5, 11, 16, 22]. The Child–Pugh class was assigned retrospectively based on the score at admission.

Patient outcome data were obtained via electronic patient record systems which are linked to death notification records and by contacting patients’ primary care physicians to confirm survival if in doubt. Outcomes of interest were survival to ICU discharge, survival to hospital discharge and survival at 12 months post-hospital discharge. Ethical approval was granted by the West of Scotland Research Ethics Committee (REC reference; 12/WS/0039).

### Statistical analysis

Parametric data were reported as mean and standard deviation and compared using Student’s t test. Nonparametric data were reported as median and interquartile range and compared using the Mann–Whitney U test. Proportions were compared using the Fisher’s exact test or Chi-squared test for association or trend, as appropriate. All *p* values calculated were two-sided, and the statistical significance threshold was <0.05.

All parameters which were under the significance threshold of <0.05 were entered into a stepwise, backward multivariate logistic regression analysis for mortality at 12 months. Receiver operating characteristic (ROC) curves were calculated for each scoring system, and the area under the curve (AUC) was determined. A threshold of AUC ≥ 0.8 for clinical utility was chosen in keeping with previous studies on this topic and statistical literature [30]. Survival analysis was conducted using the Kaplan–Meier method, from which readmissions were excluded in order to not confound the analysis. In such cases, the patient’s initial ICU admission date was used in the Kaplan–Meier analysis. Where subgroups were analysed separately, the log-rank test was used to compare the survival curves, with correction for trend if appropriate. All statistical analysis was performed using SPSS version 21 (SPSS, Inc., IBM, Chicago, USA). Advice was sought from an independent statistician.

## Results

### Demographics

Of 611 admissions screened during the study period, 84 met the diagnostic criteria for cirrhosis. Two of these admissions had missing data for a small number of variables. This meant that certain parameters, including some scoring systems, could not be calculated; however, they were included for all other analyses. The characteristics of the cohort are listed in Table 1. The mean patient age was 50.2 ± 11.2 years. Fifty-nine admissions (70.2%) were male. Fifty-six (66.7%) were from a socially deprived background. In 70 admissions (83.3%), the patient had alcohol-related disease. In 58 admissions (69.0%), the patient was receiving mechanical ventilation on arrival in the ICU. Sixty-eight (81.0%) were first admissions, and 16 (19.0%) were ICU readmissions during the same hospital stay. None of the patients admitted during the study period had been previously admitted to ICU in a separate hospital episode. The median ICU stay was 5 days (IQR 1–12.8).

**Table 1:** Clinical characteristics and predictors of 12-month mortality in 84 admissions to a general ICU with a diagnosis of cirrhosis

	All admissions (*n* = 84)	12 m survivors (*n* = 43)	12 m nonsurvivors (*n* = 41)	*p* value
Age (mean ± SD, range)*	50.2 ± 11.2	47.4 ± 10.1	53.1 ± 11.8	0.020
Male gender (*n*)	59 (70.2%)	32 (74.4%)	27 (65.9%)	0.391
SIMD quintile	1 (1–2)	1 (1–2)	1 (1–2)	0.755
Social deprivation^a^ (*n*)	56 (66.7%)	31 (72.1%)	25 (61.0%)	0.356
Alcohol-related disease (*n*)	70 (83.3%)	36 (83.7%)	34 (82.9%)	0.922
Ventilated on admission (*n*)	58 (69.0%)	30 (69.8%)	28 (68.3%)	0.884
Readmission during same hospital stay*	16 (19.0%)	13 (30.2%)	3 (7.3%)	0.008
ICU admission reason				0.114
Pneumonia	19 (22.6%)	12 (27.9%)	7 (17.1%)	
GI haemorrhage	11 (13.1%)	6 (14.0%)	5 (12.2%)	
ARDS	8 (9.5%)	4 (9.3%)	4 (9.8%)	
Sepsis	7 (8.3%)	2 (4.7%)	5 (12.2%)	
Encephalopathy	5 (6.0%)	3 (7.0%)	2 (4.9%)	
GI perforation	5 (6.0%)	0 (0.0%)	5 (12.2%)	
Trauma/burns	5 (6.0%)	3 (7.0%)	2 (4.9%)	
Decompensated cirrhosis	4 (4.8%)	0 (0.0%)	4 (9.8%)	
Seizures	4 (4.8%)	3 (7.0%)	1 (2.4%)	
Other^b^	10 (11.9%)	5 (6%)	5 (6%)	
Drug related	4 (4.8%)	4 (4.8%)	0 (0%)	
Pancreatitis	2 (2.4%)	1 (1.2%)	1 (1.2%)	
Length of ICU stay	5 (1–12.8)	7 (2–13)	5 (1–12.5)	0.243
Sodium (mEq L^−1^)	136.0 (132.0–140.8)	138.0 (135.0–142.0)	133 (131–140)	0.066
Potassium (mEq L^−1^)	3.9 (3.6–4.5)	3.9 (3.6–4.3)	3.9 (3.5–4.8)	0.809
Urea (mmol L^−1^)	8.1 (4.1–12.8)	7.1 (4.1–11.6)	9.2 (4.9–14.7)	0.123
Lactate* (mmol L^−1^)	1.9 (1.3–2.9)	1.5 (1.1–2.1)	2.4 (1.7–5.4)	<0.001
Bilirubin* (µmol L^−1^)	45.5 (20.8–108.3)	28 (14–70)	71 (41–198)	<0.001
Creatinine* (µmol L^−1^)	81.5 (57.3–162.3)	71 (57–114)	131 (63.5–197)	0.029
White cell count (×10^−9^ L^−1^)	12.4 (7.9–17)	13.3 (8.4–17.4)	7.45 (10.6–18.6)	0.466
Albumin* (g L^−1^)	20 (17–26)	23 (19–28)	18 (15.5–22)	0.002
PT ratio*	1.5 (1.2–2.0)	1.4 (1.1–1.7)	1.7 (1.5–2.5)	<0.001
Platelet count* (×10^−9^ L^−1^)	109.5 (81.5–180.8)	133 (88–215)	94 (59.5–144)	0.005
PaO_2_/FiO_2_ ratio (kPa)	21.7 (12.4–36.1)	28.3 (14.5–38.9)	18.2 (11.8–32.6)	0.070
Glasgow coma score	10.5 (3–15)	10 (5–14)	11 (3–15)	0.873
Ascites (*n*)	35 (41.7%)	14 (32.6%)	21 (51.2%)	0.083
Encephalopathy (*n*)	29 (34.5%)	13 (30.2%)	16 (39.0%)	0.397

All data are given as median (interquartile range) unless stated otherwise

*SIMD* Scottish Index of Multiple Deprivation, *GI* gastrointestinal, *ARDS* acute respiratory distress syndrome, *PT* prothrombin time, *12* *m* 12 months

* Statistically significant characteristics (*p* < 0.05)

^a^Social deprivation was defined as SIMD quintile 1 (lowest)

^b^Other include: urinary tract infection, renal failure, respiratory failure (not secondary to infection and does not meet criteria for ARDS), acute cholecystitis, biliary obstruction, diabetic ketoacidosis and post-cardiac arrest

The most common working diagnosis at ICU admission was pneumonia (*n* = 19, 22.6%) followed by gastrointestinal bleeding (*n* = 11, 13.1%), acute respiratory distress syndrome (ARDS) (*n* = 8, 9.5%), and systemic sepsis (*n* = 7, 8.3%), encephalopathy, gastrointestinal perforation and trauma, including burns (all *n* = 5, 6.0%), and decompensated cirrhosis and seizures (both *n* = 4, 4.8%).

### Long-term outcome and survival analysis

**Fig. 1:**
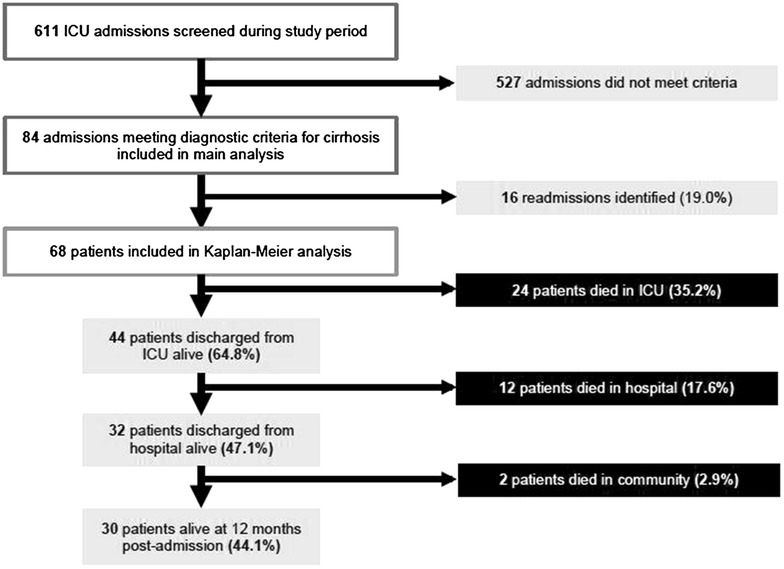
Summary of 18-month observational cohort study of patients with cirrhosis admitted to a general ICU

**Fig. 2:**
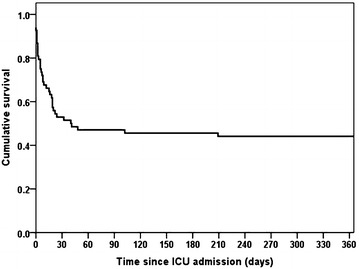
Kaplan–Meier survival curve of 68 patients with cirrhosis admitted to a general ICU

Long-term outcome data were available for all 84 admissions. After exclusion of readmissions, 68 patients were entered into the Kaplan–Meier analysis. The cumulative mortality at 12 months after ICU admission was 55.9%, with 38 deaths occurring. ICU mortality was 24 patients (35.2%), and a further 12 patients (17.6%) died in hospital after leaving ICU, giving a hospital mortality of 52.9%. Only 2 patients (2.9%) died in the community following hospital discharge. A summary figure is provided in Fig. 1, and the Kaplan–Meier curve for the whole cohort is given in Fig. 2. Subgroup analysis stratifying by the presence of alcohol-related disease or social deprivation showed no significant difference between survival curves (*p* = 0.634 and *p* = 0.537 respectively).

### Factors predictive of long-term mortality

Factors found to be predictive of mortality at 12 months in univariate analysis were increased age (*p* = 0.020), arterial lactate, serum bilirubin concentration, and PT ratio (all *p* < 0.001), serum albumin (*p* = 0.002), creatinine (*p* = 0.029) and platelet count (*p* = 0.005). Readmission during the same hospital stay was associated with increased long-term survival (*p* = 0.008). There was no significant effect observed of the presence of alcohol-related disease or levels of social deprivation on long-term survival, or any of the other clinical measurements (Table 1).

Readmission during hospital stay was excluded from the multivariate analysis as this is a subjective clinical decision highly likely to be affected by external factors. After multivariate analysis, only four variables remained statistically significant for mortality at 12 months: age (OR 1.09, 95% CI 1.03–1.15, *p* = 0.002), serum arterial lactate (OR 1.57, 95% CI 1.12–2.20, *p* = 0.010), serum bilirubin (OR 1.01, 95% CI 1.00–1.02, *p* = 0.015) and PT ratio (OR 4.82, 95% CI 1.38–16.82, *p* = 0.014). These details are given in Table 2. Based on these findings, the Child–Pugh + Lactate score was adapted to focus on the most significant components of the regression model. This was done by removing the ascites and encephalopathy components, leaving a system with a combined point score based on albumin, bilirubin and PT ratio added to the serum arterial lactate concentration. This novel score, referred to from now on as the ABC + Lactate, for Albumin, Bilirubin and Clotting (PT ratio), is shown in Table 3.

**Table 2:** Factors predictive of mortality at 12 months post-ICU admission after a backwards stepwise multivariate logistic regression analysis

	Odds ratio	95% confidence interval	*p* value
Age (years)	1.09	1.03, 1.15	0.002
Lactate (mmol L^−1^)	1.57	1.12, 2.20	0.010
PT ratio^a^	4.82	1.38, 16.82	0.014
Bilirubin (µmol L^−1^)	1.01	1.00, 1.02	0.015

*PT* prothrombin time

^a^PT ratio odds ratio is based on increments of 1

**Table 3:** Proposed ABC + Lactate score for prediction of outcome in cirrhotic patients admitted to ICU

	1 point	2 points	3 points
Albumin (gL^−1^)	>35	28–35	<28
Bilirubin (µmolL^−1^)	<35	35–50	>50
Clotting (PT ratio)	<1.7	1.7–2.3	>2.3

Lactate (mmol L^−1^) added to the total points score from above

Classification: ABC + Lactate <8 = Class 1, 8–10.9 = Class 2, ≥11 = Class 3

### Discriminative ability of scoring systems

All scoring systems studied were predictive of mortality at 12 months (*p* < 0.001 for all except Child–Pugh, *p* = 0.001). ROC curves were calculated for all scoring systems for prediction of 12-month survival, and the area under the curve values (AUC) are given in Table 4. Based on 95% confidence intervals, there were no statistically significant differences between any of the scoring systems evaluated. The only two existing systems which produced AUCs over the predetermined cut-off for clinical utility were MELD (AUC = 0.82, 95% CI 0.74–0.91) and Child–Pugh + Lactate (0.80, 95% CI 0.71–0.90). The proposed ABC + Lactate score gave the highest AUC, with a value of 0.83 (95% CI 0.74–0.92).

**Table 4:** Utility of scoring systems at predicting 12-month outcome in patients with cirrhosis admitted to ICU

	Area under ROC curve	95% confidence interval
APACHE II	0.763	0.662, 0.864
SOFA	0.748	0.642, 0.855
CLIF-SOFA	0.782	0.684, 0.880
SOFA-Lactate	0.769	0.667, 0.871
Child–Pugh	0.718	0.609, 0.828
MELD^a^	0.823	0.735, 0.911
UKELD	0.778	0.675, 0.882
RFH	0.779	0.679, 0.879
Child–Pugh + Lactate^a^	0.804	0.712, 0.896
ABC + Lactate^a^	0.831	0.744, 0.919

*APACHE* Acute Physiology and Chronic Health Evaluation, *SOFA* Sequential Organ Failure Assessment, *CLIF*-*SOFA* Chronic Liver Failure-SOFA, *MELD* Model for End-Stage Liver Disease, *UKELD* UK Model for End-stage Liver Disease, *RFH* Royal Free Hospital score, *ABC* albumin, bilirubin and clotting

^a^Models with an area under curve above the threshold of 0.8

### Comparison of classification systems

**Fig. 3:**
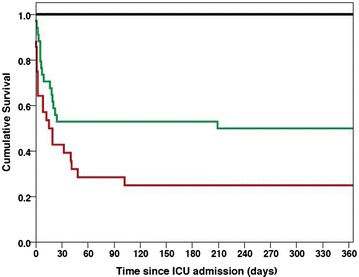
Kaplan–Meier survival curve stratified by Child–Pugh class at ICU admission. *Black lines* Child–Pugh class A (*n* = 6). *Green lines* Child–Pugh class B (*n* = 34). *Red lines* Child–Pugh class C (*n* = 28). Log-rank *p* value = 0.002

**Fig. 4:**
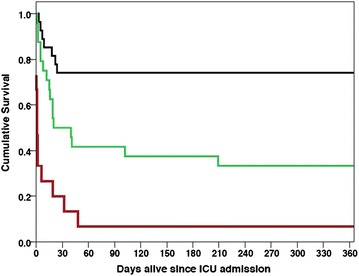
Kaplan–Meier survival curve stratified by ABC + Lactate class at ICU admission. *Black lines* ABC + Lactate class 1 (*n* = 27). *Green lines* ABC + Lactate class 2 (*n* = 24). *Red lines* ABC + Lactate class 3 (*n* = 15). Log-rank *p* value = 0.001

At the time of ICU admission, 6 patients (8.8%) had Child–Pugh class A disease, 34 (50.0%) had class B, and 28 (41.2%) had class C. Subgroup survival analysis by Child–Pugh class at ICU admission showed significant differences between the Kaplan–Meier curves (*p* = 0.002, Fig. 3): cumulative mortality at 12 months was 0.0% in patients presenting with Child–Pugh class A, 50.0% in patients with class B, and 75.0% in class C. Following its superior performance in ROC curve analysis, a classification system was also produced based on the ABC + Lactate score (Table 3), using empirical analysis of the ROC curve (not shown) to find integer cut-off points which would provide either good sensitivity or specificity. Those with an ABC + Lactate less than 8 were considered class 1 (*n* = 27, 40.9%), those scoring between 8 and 10.9 class 2 (*n* = 23, 34.8%), and those scoring 11 or higher class 3 (*n* = 15, 22.4%). Kaplan–Meier analysis by ABC + Lactate class at admission was highly significant (*p* = 0.001, Fig. 4). Cumulative mortality at 12 months was 25.9% for ABC + Lactate grade 1, 66.7% for grade 2 and 93.3% for grade 3. A comparison of Child–Pugh class and ABC + Lactate class for all three timepoints is given in Table 5.

**Table 5:** Comparison of cumulative survival by Child–Pugh class and CLIF-SOFA organ failure grade in 68 cirrhotic patients admitted to a general ICU

	Child–Pugh A (*n* = 6)	Child–Pugh B (*n* = 34)	Child–Pugh C (*n* = 28)	*p* value
Alive at ICU discharge	6 (100.0%)	24 (70.6%)	14 (50.0%)	0.015
Alive at hospital discharge	6 (100.0%)	18 (52.9%)	8 (28.6%)	0.002
Alive 12 m post-admission	6 (100.0%)	17 (50.0%)	7 (25.0%)	0.001

	ABC + Lactate1 (*n* = 27)^a^	ABC + Lactate 2 (*n* = 24)^a^	ABC + Lactate 3 (*n* = 15)^a^	*p* value
Alive at ICU discharge	24 (88.9%)	15 (62.5%)	3 (20.0%)	<0.001
Alive at hospital discharge	20 (74.1%)	10 (41.7%)	1 (6.7%)	<0.001
Alive 12 m post-admission	20 (74.1%)	8 (33.3%)	1 (6.7%)	<0.001

As readmissions were excluded so as not to confound the survival analysis, only 68 admissions are included

*ABC* albumin, bilirubin and clotting (PT ratio)

^a^2 patients had insufficient data to calculate ABC + L

## Discussion

Our findings show that cirrhotic patients admitted to ICU have high initial mortality followed by a low mortality in those patients who survive to leave hospital. In comparison with other reports of cirrhotic patients from general ICUs, our 12-month cumulative mortality of 55.9% is comparable to the figure of 59% reported by Lewis et al. and the 6-month cumulative mortality of 60% reported by Filloux et al. [3, 18] It is, however, far lower than the 90-day mortality of 76% reported by Kavli et al. [19] though this may be explained by the fact that the latter study only enrolled patients with decompensated cirrhosis and the fact that the majority of our cohort were admitted with alcohol-related cirrhosis. Compared to the contemporary literature from specialist liver ICUs, the outcome of the population of cirrhotic patients admitted to general ICUs appears to be slightly better than those requiring treatment in a specialist centre [15, 16]. As with our findings, all of these studies demonstrated a similar reduction in mortality after hospital discharge strongly suggesting that those cirrhotic patients who survive hospital have good long-term outcomes [3, 15, 16, 19].

The independent predictors of mortality in this study were serum lactate, bilirubin, PT ratio and age. Increased lactate and bilirubin have both been widely found to predict short-term mortality in critically ill cirrhotic patients [5, 11, 12, 14, 16, 21, 23], but this is the first time they have been associated with poor long-term outcome. Whilst the odds ratio of 4.82 for PT ratio appears large, this ratio represents the increased risk of mortality associated with an increase in the PT ratio of 1, a significant clinical deterioration. Surprisingly, neither alcohol-related disease nor the presence of social deprivation was found to be associated with poorer outcome, although we may have underestimated a true population effect as the vast majority of the cohort had one or both of these risk factors. In contrast to a number of previous studies [2, 14, 15, 18], mechanical ventilation at ICU admission was not found to be associated with increased mortality. Notably, readmission to ICU was a significant predictor of survival, although this may reflect a bias where patients who were readmitted to ICU were those with the highest chance of survival.

We were unable to demonstrate a statistically significant difference between the scoring systems based on ROC curve analysis; however, MELD, Child–Pugh + Lactate and ABC + Lacate all achieved an AUC higher than the predetermined threshold. However, ROC curve analysis alone is unlikely to provide a true measure of a score’s clinical usefulness [24, 31]. A classification system with mortality rates for each class is far easier for clinicians to interpret and apply at the bedside, which a score alone does not permit.

A key finding of this study is that Child–Pugh class at ICU admission stratifies patients into three groups with different long-term prognosis (Fig. 3). However, the inherent limitations of this score must be realised when interpreting this finding. Our data demonstrate that patients presenting to ICU with Child–Pugh class A disease have excellent long-term outcome, but such patients form a small proportion of critically ill cirrhotic patients—data from this study and others indicate that the vast majority of cirrhotic patients who present to ICU are Child–Pugh class B or C [11, 12, 18]. Child–Pugh class alone is unable to identify which of these class B or C patients have a sufficiently high probability of short- and long-term death to influence a decision regarding ICU admission. This limitation of Child–Pugh has been previously identified by a number of authors and may be due to a ‘ceiling’ effect of its classification system [31]. It is therefore clear that there remains a need for a bedside scoring system with the ability to identify patients with the highest risk of mortality. Whilst MELD did perform well on this cohort, it is limited by the lack of an easily accessible grading system to stratify patient into groups and hence identify patients with a high risk of mortality. Similarly, the RFH score, which has been validated for short-term outcome in both the specialist and general settings [11, 24], failed to perform as well in this long-term study. The Child–Pugh + Lactate score attempted to meet this need with the incorporation of a measure of acute illness correlated with poor short-term outcome [5]. However, this score remains limited as both the ascites and encephalopathy components are subjective and difficult to assess in the acute setting [24, 31]. Previous work from this centre has demonstrated that the omission of encephalopathy scores from both Child–Pugh and Child–Pugh + Lactate did not affect the short-term predictive value [24], and so it is perhaps unsurprising that the ABC + Lactate score, which removes these components, performed better in this study.

Unlike Child–Pugh, MELD, RFH and Child–Pugh + Lactate, the ABC + Lactate score is entirely objective and can be calculated easily at the bedside; its similarity to the well-known Child–Pugh score may also promote easy understanding amongst clinicians. In this study, ABC + Lactate classification identified a large group of patients with good long-term prognosis, and a smaller but sizeable group of patients with almost universally poor outcome (Fig. 4). ABC + Lactate class 1 patients formed 41% of the cohort and had short- and long-term outcomes similar to the general ICU population [32], whereas class 3 patients (22% of the cohort) had an ICU mortality of 75% and hospital and 12-month mortality of 93%. Crucially, ABC + Lactate was able to identify a group of patients with very high long-term mortality (class 3), suggesting it may be useful as an adjunct to the Child–Pugh classification system to identify patients at very high risk of death in the critically ill cirrhotic population.

There were some limitations to this study. The sample size is sufficiently small that an overestimation of effect bias may have occurred, particularly with some of the subgroup analyses. It should also be noted that this is a single-centre study of all-cause general ICU admissions with cirrhosis, and therefore the conclusions may not be directly applicable to other populations of critically ill cirrhotic patients. The ABC + Lactate score would require validation for both short- and long-term outcome in an external cohort of patients before it could be adopted for use in this patient population. There were no data on the number of cirrhotic patients who were referred to ICU with a very poor prognosis and therefore not accepted for admission, or those cirrhotic patients who were critically ill but were not referred for critical care. Although in our centre the vast majority of such patients are indeed admitted to ICU, this may have led the study to underestimate the mortality of critically ill cirrhotic patients by excluding those who died prior to ICU admission. Similarly, we did not formally assess decompensation on admission and therefore cannot state surely the proportion of patients who were presenting with decompensated liver disease. Finally, whilst we have assessed long-term mortality in hospital survivors as a binary outcome, we have not been able to similarly assess ongoing morbidity. It is likely that many of these patients will have significant reductions in quality of life, which whilst an important endpoint was outside the scope of this study.

## Conclusions

Patients with cirrhosis who become critically unwell should be considered for ICU admission, as whilst they have high initial mortality, those who leave hospital alive have good long-term outcomes. Child–Pugh class at ICU admission predicts outcome at 12 months but struggles to identify those patients at a very high risk of death. The novel ABC + Lactate classification system may be useful in identifying critically ill cirrhotic patients with very high long-term mortality.
